# Co-Immunization with Multimeric Scaffolds and DNA Rapidly Induces Potent Autologous HIV-1 Neutralizing Antibodies and CD8^+^ T Cells

**DOI:** 10.1371/journal.pone.0031464

**Published:** 2012-02-16

**Authors:** Juan Pablo Jaworski, Shelly J. Krebs, Maria Trovato, Dina N. Kovarik, Zachary Brower, William F. Sutton, Garrett Waagmeester, Rossella Sartorius, Luciana D'Apice, Antonella Caivano, Nicole A. Doria-Rose, Delphine Malherbe, David C. Montefiori, Susan Barnett, Piergiuseppe De Berardinis, Nancy L. Haigwood

**Affiliations:** 1 Division of Pathobiology and Immunology, Oregon National Primate Research Center, Oregon Health and Sciences University, Beaverton, Oregon, United States of America; 2 Institute of Protein Biochemistry, C.N.R., Naples, Italy; 3 Molecular and Cellular Biology Program, University of Washington, Seattle, Washington, United States of America; 4 Viral Vaccines Program, Seattle Biomedical Research Institute, Seattle, Washington, United States of America; 5 Duke University Medical Center, Durham, North Carolina, United States of America; 6 Novartis, Cambridge, Massachusetts, United States of America; University of California San Francisco, United States of America

## Abstract

To obtain proof of concept for HIV vaccines, we generated recombinant multimeric particles displaying the HIV-1 Envelope (Env) third hypervariable region (V3) as an N-terminal fusion protein on the E2 subunit of the pyruvate dehydrogenase complex of *Geobacillus stearothermophilus*. The E2 scaffold self-assembles into a 60-mer core that is 24 nm in diameter, with a molecular weight of 1.5 MDa, similar to a virus like particle with up to 60 copies of a heterologous protein accessible on the surface. Env(V3)-E2 multimers were tested alone and in combination with Env(gp160) DNA in mice and rabbits. Following two or more co-immunizations with Env(V3)-E2 and Env gp160 DNA, all 18 rabbits developed potent autologous neutralizing antibodies specific for V3 in six weeks. These neutralizing antibodies were sustained for 16 weeks without boosting, and comparable responses were obtained when lipopolysaccharide, a contaminant from expression in E. coli, was removed. Co-immunizations of Env(V3)-E2 and DNA expressing gp160 elicited moderate CD8-specific responses and Env-specific antibodies in mice. Co-immunization with DNA and E2 was superior to individual or sequential vaccination with these components in eliciting both neutralizing antibodies in rabbits and CD8^+^ T cell responses in mice. Co-immunization with DNA and multimeric E2 scaffolds appears to offer a highly effective means of eliciting rapid, specific, and sustained immune responses that may be a useful approach for other vaccine targets.

## Introduction

Despite the fact that HIV-1 utilizes highly effective mechanisms of immune evasion [1]–[3], most subjects develop both neutralizing antibodies (NAbs) and CD8^+^ T cell responses, albeit too late to clear the established infection. CD8-specific cellular immune responses contribute to early resolution of primary viremia and the maintenance of viral load [4]–[6]. NAbs can block infection in nonhuman primate models [7]–[11], and in humans can contribute to control of plasma viremia [12]. A major challenge in vaccine design has been to identify antigen presentation and delivery systems capable of eliciting strong, sustained immunity that can either prevent HIV-1 infection or provide a very high degree of control of viremia post-challenge. Vaccine approaches for HIV-1 have included recombinant viral vectors, DNA, and protein subunits, tested alone and in prime-boost combinations. These vaccines focused on eliciting cellular responses [13]–[19] following the failure of the VaxGen gp120 trial [20], [21], but T cell responses induced by adenovirus in the STEP trial were also insufficient for protection [22]. The vaccine utilized in the RV144 trial, designed to elicit both humoral and cellular responses, showed modest, transient efficacy [23]. HIV-1 virus-like particles (VLPs) or inactivated virions have elicited low-level NAbs [24]–[26] and modest protection in vaccine challenge studies [27]. Other self-assembling viral proteins such as hepatitis B surface antigen [28] or rhinovirus [29] that present key neutralization determinants from HIV have shown some promise in eliciting low-level NAbs. Cholera toxin B displaying the HIV-1 Env third hypervariable region (V3) elicited moderate cross-NAbs in rabbits [30] which may be due to its conserved structural features [31].

**Figure 1:**
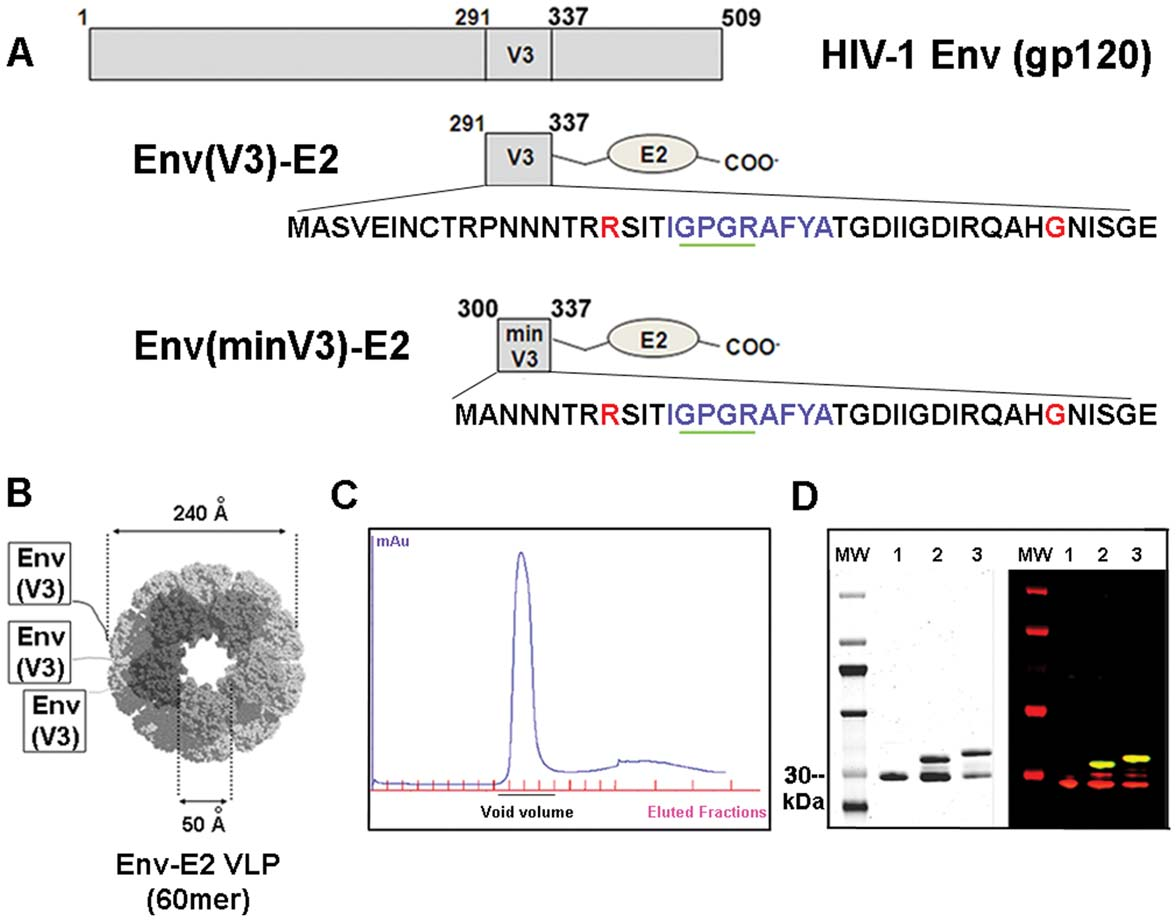
Schematic representation, purification and antigenic characterization of HIV-1 Env-E2 constructs. (**A**) Amino acid sequences of the Env (V3 and minV3)-E2 constructs are shown in relationship to gp120 (reference strain HIV-HXB2). The mAb 447-52D epitope is underlined in green and the H2^d^-restricted CTL epitope is shown in blue. Amino acid mutations are shown in red. (**B**) Schematic of the Env component displayed on the surface of the 60-mer E2 core. (**C**) S200 Sephacryl chromatograph of purified LPS-free Env(V3)-E2 particles (**D**) SDS-PAGE stained with Coomassie blue (left) and Li-Core Odyssey infrared blot (right) of E2: Lanes: MW; (1) E2 wild type (E2wt); (2) Env(minV3)-E2:E2wt hybrid particles; (3) Env(V3)-E2:E2wt 60-mers purified by gel filtration. Red in lanes 1–3 is rabbit anti-E2; green is V3 mAb 447-52D; bands that are yellow are co-stained.

We have been exploring the potential of the antigen display system E2DISP based on the acyltransferase component (E2) of the pyruvate dehydrogenase complex from *Geobacillus stearothermophilus*. E2 oligomers form 1.5 MDa 60-mer particles and are capable of displaying heterologous peptides and proteins [32]–[34]. E2 60-mer cores can be refolded from denaturing conditions *in vitro* without the help of chaperonins [34] (**Fig. 1A**). Epitopes thus displayed on the surface of the E2 core elicit both humoral and cellular immune responses [33]–[35]. More recently we have demonstrated that E2 particles displaying Gag(p17) were immunogenic in transgenic mice [36]. We evaluated the immunogenicity of Env(V3)-E2 60-mer particles in mice and in rabbits to determine whether presentation of HIV-1 V3 on E2 could focus the immune responses to the neutralization and CTL epitopes. DNA administered sequentially with viral vectors or recombinant proteins can enhance immunity, with modest levels of NAbs in rabbits [37] and control of viremia in SIV- and SHIV-challenged macaques [38], [39]. Here, we show that the co-immunization with Env(gp160) plasmid DNA and 60-mer E2 particles displaying V3 rapidly generates both NAbs and CD8^+^ T cells. Surprisingly, this strategy requires only two immunizations to derive sustained, potent responses.

## Results

### Soluble recombinant multimeric particles displaying Env V3

A DNA fragment encoding the HIV-SF162 V3 region was fused to the E2 gene in the E2DISP expression plasmid. One Cys residue (position 332) was mutated to Gly in an effort to produce homogeneous peptides with no disulfide-bonded loops (C332G) (**Fig. 1A**). The Env(V3)-E2 fusion protein initially showed 25–30% proteolysis of V3 at K305, and a K305R mutation significantly reduced proteolysis (data not shown). Two V3-E2 fusion proteins were designed; a full length V3 (291–337) and a shortened version termed minV3 (300–337) (**Fig. 1A**). The shorter min V3 was designed to enhance expression as a soluble protein, which was the method of production that was pursued prior to successful extraction from inclusion bodies. Both constructs contained the epitope recognized by the V3 NMAb 447-52D [40], a CTL epitope restricted by H2^d^ in mice, and the C332G and K305R mutations. Env(V3)-E2 and Env(minV3)-E2 monomers were expressed in *E. coli* as inclusion bodies (IB), purified, and refolded with equimolar amounts of E2 wild type (E2wt) monomers in stepdown dialysis. The resulting 60-mer particles were purified by size exclusion chromatography (**Fig. 1C**). Particles typically had more than 50 EU/ml of *E. coli*-derived LPS as a result of expression in this system, and preparations of Env(V3)-E2 with and without (<0.05 EU/ml) LPS were prepared. Resulting E2 preps were >90% pure as determined by quantitative analysis of purified protein gels (**Fig. 1D** **, left panel**). Identity was assessed by quantitative western blot (**Fig. 1D** **, right panel**). We tested the multimeric particles, separately and together, alone or in combination with HIV-1 SF162 Env (gp160) DNA, in rabbits and mice. Effects of LPS and adjuvant on the resulting immune responses were determined. Vaccine compositions and regimens tested are shown in **Table 1**.

**Table 1:** Immunization Composition and Regimen.

Animal	Group	Immunogens	LPS^a^	IFA^b^	Immunization Delivery Time (Week) and									
		Multimeric Protein	DNA			Composition: P = Protein; D = DNA								
						0	2	4	10	12	20	22	26	32
**Rabbit** c	1	Env(minV3)-E2+Env(V3)-E2	Env gp160	**+**	**+**	P		P	P			P+D	P+D	P+D
	2	Env(minV3)-E2+Env(V3)-E2	Env gp160	**+**	**+**	D		D		D	P		P	
	3	Env(minV3)-E2+Env(V3)-E2	Env gp160	**+**	**+**	P+D		P+D		P+D	P+D		P+D	
	4	Env(minV3)-E2	Env gp160	**+**	**+**	P+D		P+D		P+D	P+D		P+D	
	5	Env(V3)-E2	Env gp160	**+**	**+**	P+D		P+D		P+D	P+D		P+D	
	6	Env(V3)-E2	Env gp160	**−**	**+**	P+D		P+D		P+D	P+D			
	7	Env(V3)-E2	Env gp160	**−**	**−**	P+D		P+D		P+D	P+D			
	8	Env(V3)-E2	Env gp160	**+**	**+**	P+D		P+D				P+D	P+D	
**Mouse** d	9	None	Env gp160	**−**	**−**	D	D							
	10	Env(V3)-E2	None	**+**	**−**	P	P							
	11	Env(V3)-E2	Env gp160	**+**	**−**	P+D	P+D							
	12	Env(V3)-E2	Env gp160	**−**	**−**	P+D	P+D							
	13	Unimmunized	None	**−**	**−**	**−**	**−**							

a LPS, lipopolysaccharide.

b IFA, Incomplete Freund's adjuvant.

c 200 µg of E2 intramuscularly (IM) per animal per immunization; 36 µg of codon-optimized HIV-1 SF162 DNA intradermally via Gene Gun.

d 130 µg of E2 delivered IM; 500 µg (first dose) or 193 µg (second dose) of codon-optimized HIV-1 SF162 DNA was delivered IM.

In all cases blood samples were collected two weeks after each immunization.

### Binding antibody response in rabbits co-immunized with Env-E2 VLPs and gp160 DNA

**Figure 2:**
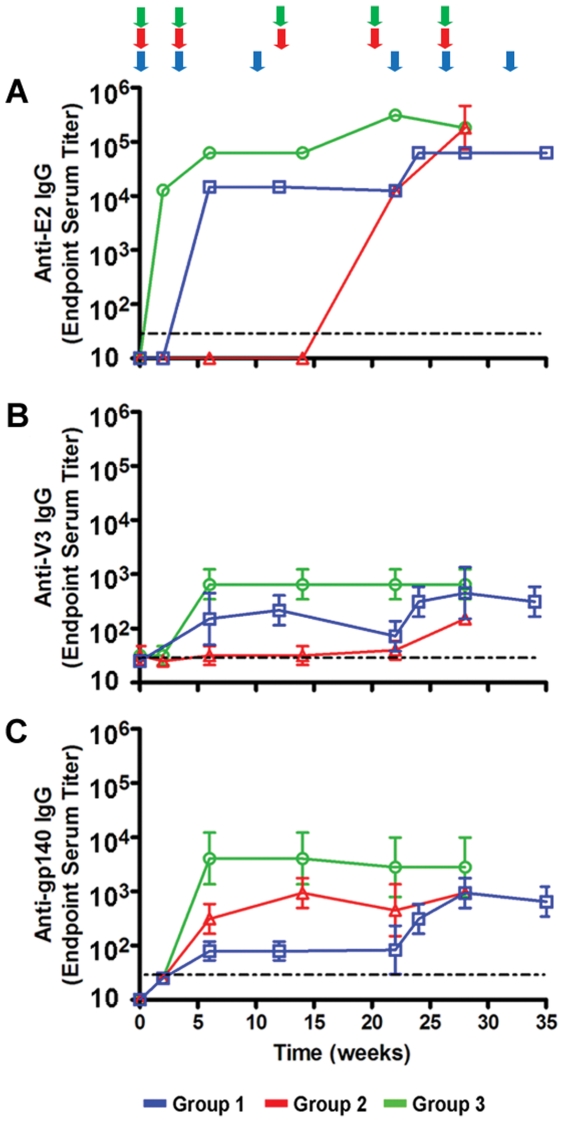
Binding antibody responses in rabbits immunized with Env-E2 particles and DNA following different immunization regimes. Binding antibody responses against E2 (E2wt) (**A**), HIV-1 Env(V3) peptide (**B**) and Env(gp140) (**C**) determined by ELISA. Data shown are geometric mean values (+/− S.D.) from rabbits in Groups 1 (blue), 2 (red) and 3 (green). Colored arrows at the top of the figure indicate the time of immunization for each group. Regimens are described in Table 1.

Three groups of New Zealand female white rabbits (n = 3 per group) were immunized with both Env(minV3 and V3)-E2 VLPs in combination with Env(gp160) plasmid DNA (DNA) using three different immunization regimens described in detail in Table 1: (i) E2-prime/E2+DNA-boost (Group 1), (ii) DNA-prime/E2-boost (Group 2) and (iii) simultaneous co-immunization with E2 and DNA (Group 3). Rabbits received 200 µg of purified E2 particles emulsified in Incomplete Freund's Adjuvant (IFA) and delivered intramuscularly (IM). DNA was delivered intradermally (ID) using the Gene gun at a dose of 36 µg. We measured binding antibodies to E2 (E2wt), V3 and gp140 (**Fig. 2**). E2 responses were seen after one and two immunizations in Groups 3 and 1, respectively (**Fig. 2A**). Rabbits in Group 2 developed E2 antibodies after the first Env-E2 boost. High titers (10^4^–10^6^) of E2 antibodies were elicited in all groups; and despite the strong E2 response, we observed the priming and boosting of Env- and V3-specific antibodies. Kinetics of V3 antibody development were similar to those of the E2 antibodies (**Fig. 2B**). The strongest V3 response was found in Group 3 and the lowest in Group 2, detected only after the second Env-E2 protein boost. Env-gp140 antibodies were seen in all three groups after the second immunization (**Fig. 2C**). Co-immunized rabbits developed the highest responses and those receiving Env-E2 alone had the lowest response. The addition of DNA in the immunization regimen of this last group boosted the level of antibodies to gp140. Immunization with DNA alone (Group 2) was sufficient to elicit gp140-specific antibodies after two and three immunizations; boosting with Env-E2 in these animals did not increase titers. In all cases the strongest and most rapid antibody responses were observed in Group 3 following co-immunization with DNA and Env-E2 (**Fig. 2**).

### Rapid induction of autologous NAbs in rabbits

**Figure 3:**
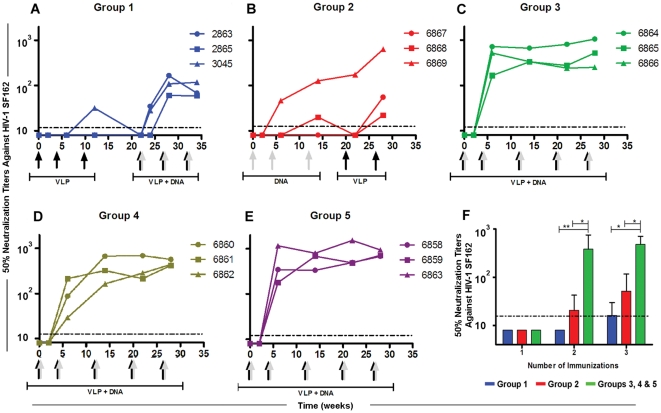
Neutralizing antibody responses in rabbits immunized with Env-E2 particles and DNA following different immunization regimes. (**A–E**) Lines indicate NAbs *versus* HIV-SF162 in each rabbit in Groups 1 (blue), 2 (red), 3 (green), 4 (golden) and 5 (purple). Arrows at the bottom of each graph indicate time and type of immunization: grey arrows, DNA vaccination; black arrows, Env-E2 VLPs; combined grey and black arrows, co-administration of DNA plus E2. (**F**) NAb titers during priming stages for three immunization regimens: (i) Env-E2 (Group 1, in blue), (ii) DNA (Group 2, in red) and (iii) co-administration of Env-E2 plus DNA (Groups 3, 4 and 5; in green). Bars are mean titer values (+S.D.) in each group. Asterisks denote statistical significance: ** P<0.01; * P<0.05. Dotted lines indicate the limit of detection of the assays.

We measured NAbs against the autologous HIV-1 SF162 pseudovirus using the TZM-bl assay [41]. Only one rabbit immunized with Env-E2 alone developed weak NAbs and only after the third immunization (#3045 NAb_w12_ = 32) (**Fig. 3A** **, VLP priming stage**). To increase responses in the DNA primed rabbits [42], we boosted them with a combination of Env-E2 and Env(gp160) DNA at weeks 22, 26, and 32. Co-immunization with E2 and DNA increased the level of NAbs in all rabbits after two inoculations (**Fig. 3A**). After the third co-immunization, titers did not increase. Using the same vaccine components in a DNA-prime/protein-boost immunization regimen (Group 2), only one rabbit developed significant NAbs during the DNA-priming stages (**Fig. 3B**). The NAb level of a second rabbit was measurable only transiently above the limit of detection at week 12. However, boosting with Env-E2 alone increased NAb titers in all three animals from this group (**Fig. 3B**). Two of the three animals with negligible responses generated detectable NAbs after boosting. Mean NAb titer at week 28 was 237, and the highest responder had a titer of 634.

**Figure 4:**
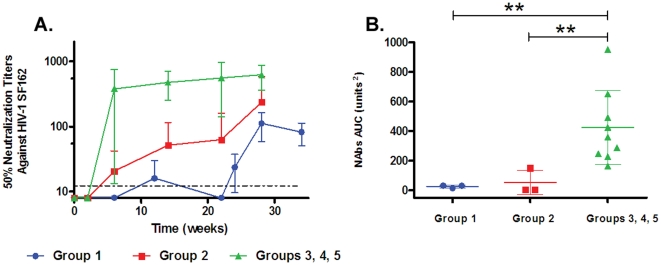
Neutralization of HIV-1 SF162 by sera from rabbits co-immunized with HIV-1 Env-E2 VLPs and Env(gp160) plasmid DNA following different immunization regimens. (**A**) Neutralizing activity in the sera of rabbits from Groups 1 (blue), 2 (red) and 3–5 (green) against HIV-1 SF162. Final titers were calculated as the reciprocal of the dilution of serum necessary to inhibit infection by 50% and informed as the mean titers (+/− S.D.) for each group. The dotted line represents the limit of detection of the assay (1∶16). (**B**) Comparison of the NAb area under the curve (AUC) among the above mentioned groups. Asterisks denote statistical significance: ** P<0.01.

Rabbits co-immunized with both Env-E2 VLPs and Env(gp160) DNA (Group 3) rapidly developed a potent autologous NAb response (**Fig. 3C**). All animals consistently generated high levels of NAbs after only two immunizations within a period of six weeks (Mean NAb_w6_ = 466 and Max NAb_w6_ = 714). Moreover, the level of NAbs in all animals from this group remained fairly constant with boosts over a period of 28 weeks (Mean NAb_w28_ = 609 and Max NAb_w28_ = 1058). To determine which Env-E2 VLP was more effective in eliciting NAbs, each construct was tested separately and co-administered with DNA (Groups 4 and 5, **Table 1**). NAbs levels generated by Env(minV3)-E2 (**Fig. 3D**, Group 4) and Env(V3)-E2 (**Fig. 3E**, Group 5) given separately (co-administered with DNA) were not statistically different from those obtained when both Env-E2 preparations were combined (Group 3). Since equivalent results were obtained with the full length V3 construct, this construct was used for additional experiments. To summarize, no NAb responses were observed for any of the three regimens after the first immunization (**Fig. 3F**). After the second and third immunization weak and variable levels of NAbs were detected in animals inoculated with VLP or DNA (Groups 1 and 2). In contrast, co-immunization with multimeric 60-mers and DNA elicited significantly higher levels of NAbs than E2 particles or DNA alone (*P*<0.05), after two and three immunizations, better than either of these components delivered alone or sequentially. Area under the NAb titer curve (AUC) for the co-immunization regimen (Groups 3–5, n = 9) was significantly higher compared to that for either sequential regimen (*P*<0.01; **Fig. 4**).

### V3 specificity of NAbs

Neutralization of HIV-SF162 in sera from rabbits from all of the groups was specifically inhibited when samples were pre-incubated with the SF162 V3 peptide, but not with a V3 scrambled peptide (**Table 2**). Sera from the highest responding rabbits from Groups 1 and 2 were included to compare the responses generated by the sequential regimens. Neutralization in rabbit 3045 (Group 1) showed a 67% V3 peptide inhibition after receiving three Env-E2-only immunizations and three Env-E2+DNA immunizations. Neutralization in the serum from rabbit 6869 (Group 2) was inhibited >90% by the V3 peptide at both weeks 14 and 28 (pre and post protein boost, respectively). NAbs from all rabbits from Group 3 were specifically inhibited by the V3 peptide >95% after only two immunizations and was maintained until week 28. There were no significant differences among samples from the different groups. Low NAb levels in the other samples from Groups 1 and 2 prevented us from obtaining reliable peptide competition data.

**Table 2:** HIV-SF162 neutralization competition assay for V3 specificity.

	ID_50_ in TZM-bl Assay^1^ (HIV-SF162)					
Group	Rabbit ID	Bleed week	No peptide	Scrambled V3 peptide	V3 peptide	Percent inhibition
1	3045	34	118	118	40	67
2	6869	14	126	222	17	92
		28	634	634	8	99
3	6864	6	714	1093	8	99
		28	1058	963	29	97
	6865	6	166	204	8	96
		28	519	660	43	93
	6866	6	519	820	8	99
		28	251	206	60	71
4	6860	14	674	1143	143	87
		28	568	669	66	90
	6861	14	323	647	42	94
		28	420	251	25	90
	6862	14	164	215	52	76
		28	442	327	67	80
5	6858	6	346	734	8	99
		28	685	372	42	89
	6859	6	180	314	8	97
		28	709	362	66	82
	6863	6	1170	2268	8	100
		28	944	839	16	98

1 *Values are the serum dilution at which relative luminescence units (RLUs) were reduced 50% compared to virus control wells (no test sample).*

### Similar responses observed without adjuvant using LPS-free Env-E2 particles

**Figure 5:**
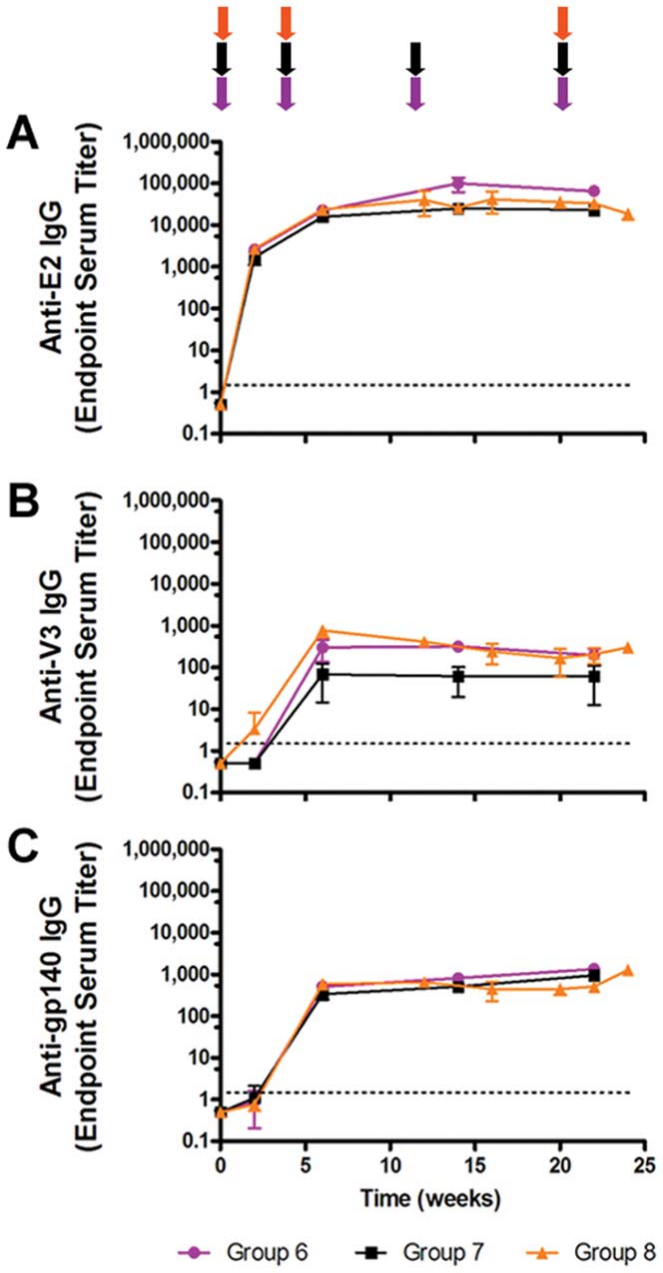
Binding antibody responses in rabbits co-immunized with Env(V3)-E2 particles and DNA with and without LPS and IFA. Specific antibody responses against E2 (E2wt) (A), HIV-1 Env(V3) peptide (B) and Env(gp140) (C) determined by ELISA. Data shown are geometric mean values (+/− S.D.) from rabbits in Groups 6 (purple), 7 (black) and 8 (orange). Colored arrows at the top of the figure indicate the time of immunization for each group.

**Figure 6:**
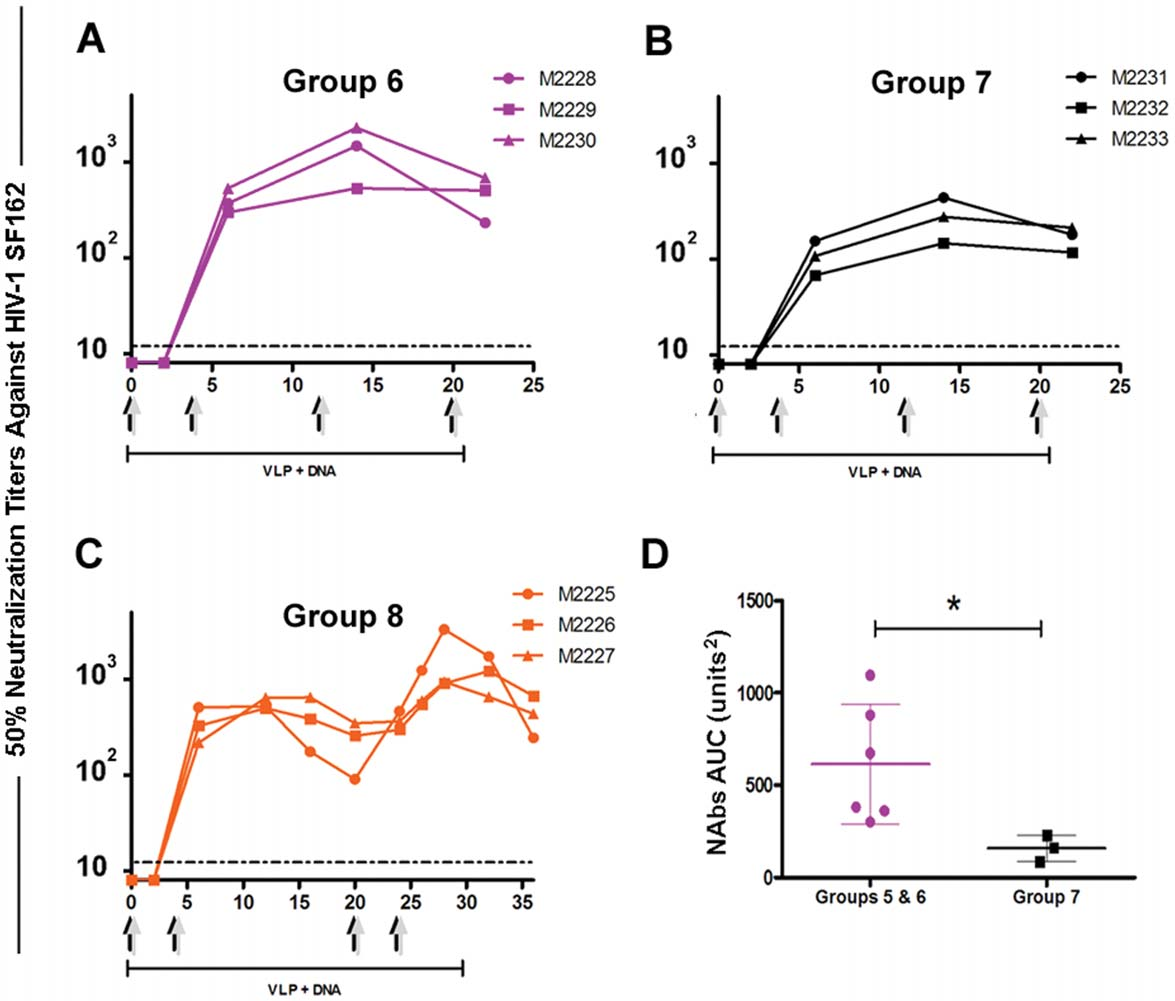
Neutralizing antibody responses in rabbits co-immunized with Env(V3)-E2 particles and DNA with and without LPS and IFA. (**A–C**) NAbs against HIV-SF162 in sera from rabbits immunized with Env(V3)-E2 co-administered with Env(gp160) DNA as described in Table 1, Groups 6–8. Lines indicate NAb titers for individual rabbits in each group: (A) Group 6, LPS-negative Env(V3)-E2, plus IFA; (B) Group 7, LPS-negative, no IFA; (C) Group 8, LPS-positive, plus IFA. Arrows at the bottom on the figure indicate time and type of immunization. The dotted line represents the limit of detection of the assay (1∶16). (**D**) Comparison of the NAb area under the curve (AUC) between groups immunized following similar regimens and using same immunogens with (Groups 5 and 6) or without the addition of IFA (Group 7).

To examine the effect of LPS on the immune responses, we co-immunized a group of rabbits with LPS-free Env(V3)-E2 particles and Env(gp160) DNA with and without IFA (Groups 6 and 7). Binding antibodies directed to E2wt, V3 peptide, and gp140 were similar in both timing and magnitude using LPS-free E2 proteins (**Fig. 5**) compared to LPS-containing preparations when co-immunized with DNA (**Fig. 3**). Following the same inoculation schedule as Group 5, after two immunizations the NAb titer was 400 (Group 6, **Fig. 6A**), and although the NAb titers increases after the third co-immunization (Mean NAb_w12_ = 1679), they were not significantly different than those observed in rabbits co-inoculated with LPS-contaminated Env(V3)-E2 (Group 5; **Fig. 3E**). Three rabbits (Group 7) were co-immunized with LPS-free Env(V3)-E2 and Env(gp160) DNA, without the addition of IFA to the protein. After two immunizations, the NAbs rapidly increased in all rabbits to a mean NAb_w6_ = 110 (**Fig. 6B**). After the third and fourth inoculations, NAbs increased (Mean NAb_w14_ = 171), but did not reach levels observed in rabbits receiving IFA. NAbs in Group 7 without addition of adjuvant were significantly lower than in Groups 5 and 6 (*P*<0.05; **Fig. 6D**). Thus IFA contributes to elevating the NAb response. To examine the duration of the NAb response, rabbits were co-immunized twice with Env(V3)-E2 particles and Env(gp160) DNA (Group 8) at weeks 0 and 4, followed by a rest until week 20. Binding antibodies had similar kinetics as seen in other groups and were sustained for at least 16 weeks following the last boost at week 4 (**Fig. 6C**). After two immunizations the mean NAb titer was 352, similar to titers in Group 5 (mean titer 565). Levels of NAbs increased at eight weeks after the last immunization but showed a decreasing trend at 12 weeks for one rabbit, and at 16 weeks for all three rabbits (Mean NAb_w16_ = 474). Boosting at week 20 restored the NAbs to the prior level. These results confirmed the rapid development of high titer NAbs in all rabbits co-immunized with Env-E2 and Env(gp160) DNA, with a duration of at least three months from the last immunization.

### Minimal breadth of NAbs elicited through vaccination with Env(V3)-E2 plus DNA

A subgroup of samples was selected from weeks 6 and 28 in Groups 3 and 5 to measure heterologous neutralization. The only significant responder from Group 2 (rabbit 6869) was included. Sera were assayed in the TZM-bl assay against a panel of 12 Clade B pseudoviruses with differing degrees of sensitivity (Tier 1A: SF162 and MN.3; Tier 1B: BaL.26, SS1196.1, 6535.3, and BZ167.12; and Tier 2: QH0692.42, PVO.4, RHPA4259.7, WITO4160.33, REJO and CAAN5342.A2) [41]. Murine leukemia virus (MLV) and a pre-immune sera pool were used as negative controls. A weak to moderate level of neutralization of a subset of Tier 1A viruses was observed (**Table 3**). Sera from all animals neutralized SF162.LS and most neutralized the MN.3 with titers ranging from 23 to 3922. The highest response was observed in week 6 sera for animal #6863 from Group 5, co-immunized with E2 and DNA. Sera from only two rabbits neutralized BaL.26. Rabbit #6863 neutralized BaL.26 at weeks 6 and 28. Rabbit #6864 from Group 3 neutralized BaL.26 weakly and only with serum obtained at week 28. Interestingly, serum from rabbit #6863 weakly neutralized three additional Clade B isolates: Tier 1B virus SS1196.1, and Tier 2 viruses QH0692.42 and REJO (titers<75). No neutralization was observed against any of the other pseudoviruses tested.

**Table 3:** Neutralization of HIV-1 Pseudoviruses.

ID_50_ in TZM-bl^1^						
Group	Rabbit ID	Bleed Week	MN.3	SF162.LS	Bal.26	SVA-MLV
2	6869	14	24	258	–	–
		28	–	444	–	–
3	6864	6	287	801	–	–
		28	389	960	23	–
3	6865	6	–	136	–	–
		28	45	427	–	–
3	6866	6	338	556	–	–
		28	140	339	–	–
5	6858	6	–	532	–	–
		28	23	598	–	–
5	6859	6	25	343	–	–
		28	117	684	–	–
5	6863	6	1884	3922	41	–
		28	763	1290	35	–
Pre-bleed	Pool	0	–	–	–	–

1 *Values are the serum dilution at which relative luminescence units (RLUs) were reduced 50% compared to virus control wells (no test sample).*

*– indicates no neutralization seen at a dilution of 1∶20 (<20).*

### CD8+ T cells induced in mice co-immunized with Env(V3)-E2 particles and Env(gp160) DNA

**Figure 7:**
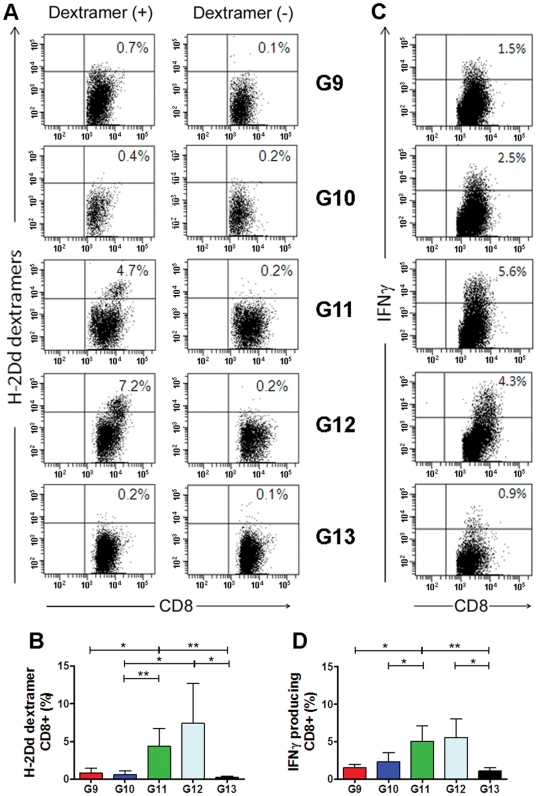
Cellular immune responses in mice immunized with HIV-1 Env(gp160) plasmid DNA, Env(V3)-E2 VLPs, or the combination of both. Cellular immune responses were measured using two different assays: (i) Dextramer analysis of antigen-specific CD8+ T cells and (ii) IFN-γ intracellular cytokine staining (ICS). (**A**) Representative dextramer analysis results from a single individual in each group (9–13). The number in the upper right of each square represents the percentage of dextramer-positive cells after gating on CD8+ cells. (**B**) Mean H2D^d^ dextramer analysis values (+S.D.) of antigen-specific CD8^+^ T cells from all mice in each group (n = 3). (**C**) INF-γ production in CD8+ gated cells from a single representative mouse from each group. The percentages of IFN-γ positive cells are indicated in the upper right corner of each square. (**D**) Mean (+S.D.) of percentage values of IFN-γ secreting CD8^+^ T cells from all mice in each group (n = 3). Asterisks denote statistical significance: ** P<0.01; * P<0.05.

As shown in **Table 1**, five groups of BALB/c mice (n = 3 per group) were immunized twice IM with HIV-1 SF162 Env(gp160) DNA alone (Group 9), Env(V3)-E2 alone (Group 10), or by co-administration of the DNA plus Env(V3)-E2 (Group 11). DNA and proteins were delivered IM at different sites. A fourth group received the same co-immunization with Env-E2 and DNA using LPS-free Env(V3)-E2 particles described above (Group 12). Group 13 (unimmunized mice) served as controls. Ten days after the second immunization, mice were sacrificed and splenocytes were isolated and re-stimulated with LPS-induced blast cells pulsed with V3 peptide. After 6 days of *in vitro* stimulation, effector cells were tested for V3 peptide-specific responses by dextramer T cell staining analysis and intracellular cytokine staining (ICS). A representative set of dextramer T cell staining obtained by flow cytometry for splenocytes isolated from each group of immunized mice is illustrated in **Fig. 7A**. Results obtained from all animals are summarized and statistically analyzed in **Table 4** and **Fig. 7B**, respectively. A significantly higher percentage of CD8^+^ splenocytes from co-immunized mice (Group 11) stained positive for the V3 dextramer, compared to mice immunized with plasmid DNA or VLP alone. Likewise, 7.43% of splenocytes (mean value) specifically stained positive for the V3 dextramer in mice that were co-immunized with LPS-free VLPs (Group 12). These results suggest that co-administration of DNA and Env-E2 VLPs was required to induce *in vivo* peptide-specific CD8^+^ T-cells able to recognize the class I MHC IGPGRAFYA peptide. Splenocyte-derived CD8^+^ T cells were assessed for IFN-γ production by intracellular cytokine staining, as shown in a representative example **(** **Fig. 7C**). As seen with the dextramer staining, CD8^+^ T-cell activation in response to the antigenic MHC class I peptide and CD8^+^ T-cell response to the Env proteins V3 specific. As observed with dextramer staining, co-immunized mice (group 11) displayed a significantly higher fraction of CD8^+^ T cells producing IFN-γ (**Fig. 7D** **and** **Table 4**) in comparison to DNA (Group 9) or E2 (Group 10) alone. Comparable results were obtained using the LPS-free Env-E2. T cell responses elicited by each of the components (DNA or Env- E2) separately did not differ from levels in non-immunized mice, underscoring the effectiveness of the combination of the two components to elicit an enhanced immune response. Binding antibodies directed to E2, V3, and Env gp140 and low-level NAbs to SF162 were detected in sera from mice immunized with Env(V3)-E2 alone or with DNA (Table 5, Groups 10 and 11), with some mice responding better than others.

**Table 4:** H2^d^-Dextramer Staining and Intracellular Cytokine Staining of CD8^+^ T Cells from Immunized Mice.

			Dextramer Staining		ICS^a^ for IFN-γ					
Group	Immunization	Mouse number	Experiment #1^c^	Experiment #2^c^	Mean	SD^b^	Experiment #1^c^	Experiment #2^c^	Mean	SD^b^
**9**	DNA	1	0.2	–	0.77	0.67	1.3	–	1.53	0.40
		2	0.6	–			2.0	–		
		3	1.5	–			1.3	–		
**10**	E2	4	0.2	0.2	0.62	0.46	1.1	1.7	2.32	1.21
	LPS-positive	5	0.7	1.3			4.6	1.9		
		6	0.3	1.0			2.5	2.1		
**11**	DNA+E2	7	6.6	4.5	4.35	2.36	6.1	3.8	5.06	2.06
	LPS-positive	8	4.0	2.2			5.6	3.8		
		9	7.4	1.4			8.4	2.7		
**12**	DNA+E2	10	–	7.0	7.43	5.26	–	4.4	5.56	2.46
	LPS-free	11	–	12.9			–	8.4		
		12	–	2.4			–	3.9		
**13**	Unimmunized	13	0.2	0.2	0.23	0.16	0.7	1.8	1.13	0.37
		14	0.3	0.0			0.9	1.2		
		15	0.2	0.5			1.1	1.1		

a ICS, intracellular cytokine staining;

b SD, standard deviation;

c Values are expressed as percentages (%) minus the average percentage of the cells with medium alone; LPS, lipopolysaccharide; –, not done.

**Table 5:** Binding and neutralizing antibodies in immunized mice.

			Anti-E2 Ab titer^a^	Anti-V3 Ab titer^a^	Anti-gp140 Ab titer^a^	ID_50_ in TZM-bl^a^				
Group	Immunization	Mouse number	Experiment #1	Experiment #2	Experiment #1	Experiment #2	Experiment #1	Experiment #2	Experiment #1	Experiment #2
**9**	DNA	1	–	–	–	–	–	–	–	–
		2	–	–	–	–	–	–	–	24
		3	–	–	–	–	–	–	–	–
**10**	Env(V3)-E2	4	435,915	211,306	4	6	–	–	–	40
	LPS-positive	5	2,864,614	457,696	4,014	463	20	569	22	54
		6	206,497	252,943	859	11	287	6	–	31
**11**	DNA+Env(v3)-E2	7	268,190	87,161	270	10	93	98	–	24
	LPS-positive	8	105,977	199,816	11,582	175	64	314	32	59
		9	319,674	131,605	440	721	27	464	–	93
**13**	Unimmunized	13	–	–	–	–	–	–	–	–
		14	–	–	–	–	–	–	–	–
		15	–	–	–	–	–	–	–	–

a Values are endpoint serum titers with samples from two weeks post final immunization; –indicates not detected; LPS, lipopolysaccharide.

## Discussion

In this study we produced HIV-1 Env-based E2 60-mer particles and evaluated their immunogenicity alone and in combination with an Env(gp160) expression plasmid. Previously, E2 particles displaying HIV-1 Gag were shown to bear helper T cell epitopes and to elicit CTL and antibody responses directed to HIV-1 Gag-p17 in HLA-A1 transgenic mice [36]. The responses observed after co-immunization with Env(gp160) DNA were characterized by peptide-specific Class I-restricted CD8^+^ T cells in BALB/c mice, and the rapid elicitation of high, sustained levels of NAbs in rabbits that was V3-specific. Simultaneous co-administration of this multimeric protein and DNA resulted in rapid, high, sustained immunity that was of greater magnitude than administering the individual components alone or in a sequential DNA-prime/protein-boost regimen.

Antibody titers directed to the E2 core were rapidly induced, as was the development of antibodies targeting to the HIV-1 Env epitope presented on the scaffold. All nine rabbits receiving both Env(V3)-E2 scaffolds together with Env(gp160) DNA following any of the three different immunization regimens (Groups 1–3) developed specific antibodies targeting Env. Our results show that Env(gp160) DNA is more effective in eliciting specific antibodies targeting the whole gp140 protein, whereas Env(V3)-E2 proteins are more effective at focusing the response to the V3 peptide presented on the E2 surface. The individual contributions of each of these components can be complemented by the other following sequential administration as shown for Groups 1 and 2. The effect is clearly potentiated when both components are administered together as is the case of Groups 3–8, since all 18 animals from those groups developed the highest responses to both V3 and gp140, and these responses were sustained during the entire immunization schedule.

Intramuscular immunization of rabbits with Env(minV3)-E2, Env(V3)-E2, or both, co-administered with HIV-1 Env(gp160) DNA delivered ID consistently elicited strong NAb responses after only two immunizations (by week 6), levels typically not seen until after 20^+^ weeks and many more immunizations [43], [44]. These high levels were sustained with three additional inoculations for a period of seven months, when the experiment was terminated. The NAbs were maintained for at least three months during a gap in immunizations (Group 8). Co-immunization of the Env-E2 plus DNA elicited 100-fold higher NAb responses compared to those elicited by each component individually (*P*<0.05). Following as few as three immunizations, this combination protein plus DNA approach elicited a stronger NAb response than a more standard DNA-prime/protein-boost regimen. The V3-E2 constructs, not surprisingly, focused the immune responses to the V3 epitope displayed on the surface. The V3 constructs tested were not effective in generating broadly NAbs; however, we observed a modest level of neutralization of some Tier 1 viruses. It is possible that constrained V3 peptides might be more effective in this context in eliciting broader responses than we observed, as seen with recombinant CTB displaying V3 [45].

Env-E2 particles delivered IM co-administered with Env(gp160) DNA delivered IM at a different sites elicited V3-specific CD8^+^ cellular immune responses in mice. As was seen in rabbits with antibodies, the CD8 response was significantly higher compared to that elicited by E2 or DNA when they were administered individually, where responses were only rarely distinguishable from the non-immunized control group. These results suggest that both the E2 and DNA components are important in inducing both cellular and humoral immune responses. This observation is in agreement with previous observations [33], [35], [36] demonstrating that antigens displayed in the E2DISP system can be cross-presented in a MHC Class I context by antigen presenting cells (APCs). The efficacy of DNA immunization in eliciting cellular immunity in mice is well established [46]. Antigen presenting cells (APC) can phagocytyze these transfected somatic cells and the antigen of interest can be either cross-presented on MHC Class I molecules or presented by MHC Class II, inducing CD4^+^ T-cell responses [47].

One of the theoretical advantages of DNA vaccines is their ability to express the native proteins and particles *in vivo*, preserving conformation-sensitive epitopes in the context of cellular antigens as would take place during virion production. Immunogens that are capable of preserving native HIV-1 Env trimeric structure are thought to elicit better NAb responses [48]. The robust antibody responses elicited with this regimen suggest that DNA can effectively prime antigen-specific B cells that are specifically boosted upon administration of protein. This principle is consistent with the concept of immune focusing [31], [49], driving antibody responses toward important structural domains without diverting the response to immunodominant, but ultimately unimportant, regions of Env. In this study, we used a V3 unconstrained peptide as a model to test the ability of the E2DISP system to elicit NAbs and cellular immunity. This model protein does not directly address the importance of the priming with native Env-expressing DNA. We are currently utilizing this system to focus immunity on other conserved regions of Env such as the CD4 binding site and the membrane proximal external region (MPER).

It has been proposed that CpG motifs present in DNA trigger toll-like receptors (TLRs) and thereby stimulate immunity. In this study we demonstrated the intrinsic immunogenicity of the E2 particles, evidenced in the rapid anti-E2 antibody responses after a single immunization. Removing LPS from Env-E2 formulations did not diminish immunity, indicating that immunogenicity was neither enhanced nor diminished in the presence of LPS. Taken together these properties of both DNA and E2-VLPs could, in part, explain the potentiation of the immune response that we observed in the present study. Further experiments are needed to understand the mechanisms of the co-immunization methods that we have used. Combining the properties of these two antigen delivery systems may have the effect of maximizing the efficiency of antigen presentation and more effectively engaging both arms of the adaptive immune response. Repetitive presentation of an epitope, as with E2 particles, can induce a stronger immune response by triggering oligomerization of B cell receptors recognizing the epitope [50]–[52]. The E2DISP delivery may be particularly efficient at utilizing this mechanism, given its potential to display up to 60 copies of antigen on the surface of each particle. More importantly, this system is capable of displaying multiple heterologous peptides or proteins per particle. The long term potential of this system is that low cost vaccines for HIV and other disease applications could be designed to generate both CD8+ T cell responses and antibodies.

Altogether, these results suggest that this antigen presentation system, coupled with the simultaneous DNA and E2 particle immunization regimen, may hold significant hope for effective immunogenicity of HIV and other vaccines in the clinic.

## Materials and Methods

### Rabbits and mice

All rabbit studies were performed in accordance with the standards outlined by the National Institutes of Health Guide for the Care and Use of Laboratory animals. The ONPRC is an AAALAC-accredited institution. Rabbit studies were performed according to the rules approved by the Institutional Animal Care and Use Committee (IACUC) at the Oregon Health & Science University, protocol no. 0825. All mouse experiments were carried out in accordance with European Union Laws and Guidelines for the Care and Use of Laboratory Animals and were approved by the Institutional Review Board and performed according to rules approved by the Animal Ethics Committee (permission no. 137/2006-A). Female New Zealand White rabbits (Western Oregon Rabbit Company, Philomath, OR) were housed at the Oregon National Primate Research Center (ONPRC) at Oregon Health & Science University. Eight-week-old female BALB/c mice (H-2^d^ MHC) were obtained from the Charles River Laboratory (Lecco, Italy). Animals were housed under specific pathogen free conditions at the Animal Facility of the CNR, Naples, Italy.

### Rabbit immunizations

New Zealand White female rabbits were immunized intramuscularly with 200 µg total protein per immunization with and without Incomplete Freund's adjuvant (IFA) (outlined in Table 1). Codon-optimized SF162 gp160 DNA was delivered intradermally via Gene Gun (Bio-Rad) at a pressure of 400 psi. A total of 36 mg of DNA was delivered in 18 shots of 2 mg DNA each given in clusters of three non-overlapping positions at six shaven sites (lower back, inside of back legs, and abdomen) as previously described [39]. Blood was collected two weeks after each immunization; the serum was separated and heat inactivated at 56°C for 1 h before being stored at −20°C.

### Mouse immunizations

Four different groups of three BALB/c mice were intra-muscularly immunized twice following four different immunization regimens (Table 1): at day 0 they received (i) 500 µg/dose of DNA (pEMC* encoding HIV-1 SF162 gp160) (group 9), or (ii) 130 µg/dose of Env(V3)-E2 (group 10), or (iii) the co-administration of both DNA and VLP, delivered into different mouse legs (group 11), or (iv) the co-administration of DNA and LPS-free VLPs (group 12). Twelve days after the first immunization, mice were boosted IM by injecting 193 µg/dose of pDNA and the same doses described above for the proteins. A final group was not immunized and used as a control (group 13). Two separate experiments were performed: groups 9, 10, 11, 13 were included in the first set of experiments and groups 10, 11, 12, 13 in the second set of experiments. Ten days post boosting, blood was collected from the periorbita for immunological analysis and mice were sacrificed. Single-cell suspensions of splenocytes isolated from BALB/c mice were co-cultured at a density of 2.5×10^6^ cells/ml with IGPGRAFYA_311–318_ peptide-pulsed γ-irradiated (10,000 rad) lipopolysaccaride-blasts (1.25×10^6^ cells/ml LPS-blasts) produced from non-immunized BALB/c mouse. Ag-pulsed LPS blast cells consisted of splenocytes that were cultured in RPMI 1640, in the presence of 25 µg/ml LPS (Sigma), supplemented with 10% FCS, 5×10^−5^ M 2-ME, 1 mM glutamine, 1 mM sodium pyruvate, and 7 µg/ml dextran sulfate (Sigma) for 3 days and pulsed for 3 h with 10 µg/ml of IGPGRAFYA_311–318_ peptide. After 6 days of co-culture, effector cells were harvested and assayed for dextramer staining and Intracellular cytokine IFNγ staining (IFNγ-ICS).

### Construction of HIV-1 ENV-E2DISP plasmids

The Env(V3)-E2 and Env(minV3)-E2 expression vectors were constructed from the previously described pETE2DISP plasmid [34].The oligonucleotide sequence encoding the SF162 Env V3 loop peptide 291–336 was cloned into the pETE2DISP vector for expression of the Env peptide as an N-terminal fusion to the E2 core scaffold (Fig. 1A, B). The oligonucleotide sequence encoding V3 was amplified using primers GGCGGCGGCCCATGGCCTCTGTAGAAATTAATTCTAC and GGCGGCGGCCCCGGGTTCTCCACTAATGTTACAATG containing the restriction sites *NcoI* and *XmaI* (New England Biolabs). Cycling conditions for the PCR were as follows: denature at 94°C for 2 min, 10× (94°C for 15 sec, 49°C for 30 sec, and 72°C for 60 sec), 20× (94°C for 15 sec, 65°C for 30 sec, and 72°C for 60 sec), and a final elongation of 72°C for 7 min. The PCR product and the pETE2DISP vector were double digested with *NcoI* and *XmaI* and ligated together with T4 DNA ligase (New England Biolabs) before transformation into BL21 (DE3) CodonPlus-RIPL cells (Stratagene). To decrease proteolytic degradation of Env(V3)-E2, a K305R mutation was introduced using primer CTAACAATAATACAAGAAGAAGTATAACTATAGGACCGG. Likewise, to decrease intramolecular bonding and increase solubility of Env(V3)-E2, a C332G mutation was introduced using the primer GAGATATAAGACAAGCACATGGCAACATTTAGTGGAGAACC. Both mutations were generated using the QuikChange Multi Site-Directed Mutagenesis kit (Stratagene), per manufacturer's instructions. The minimized V3 construct Env(minV3_C332G/K305R_)-E2 was generated from the corresponding full-length Env(V3_C332G/K305R_)-E2 construct via deletion of amino acids 291–299 using the QuikChange II Site-Directed Mutagenesis (Stratagene) and primer CCATGGCCTCTGTAGAAATTAATTGTACCATGGCTAACAATAATACAAGAAGAAGTATAAC, which contains an *NcoI* site corresponding to residue 299. In-frame ligation of all constructs was confirmed by sequencing. For simplicity, these constructs Env(V3_C332G/K305R_)-E2 and Env(minV3_C332G/K305R_)-E2 are annotated in this document as Env(V3)-E2 and Env(minV3)-E2, respectively.

### Expression, purification and refolding of Env-E2 multimeric particles

Plasmids encoding the E2wt and HIV-1 Env-E2 fusion proteins were maintained and expressed after induction with Isopropyl b-D-1 thiogalactopyranoside (IPTG) in *Escherichia coli* strain BL21 (DE3) CodonPlus-RIPL cells (Stratagene). The soluble E2wt-containing fraction was recovered from E. coli lysates after centrifugation at 10,000× g for 10 min at 4°C and was purified using a Sephadex G-25 column for buffer exchange (GE Healthcare), a Detoxi-Gel for LPS removal (Pierce), and a Q-Sepharose anion exchange column (GE Healthcare). Peak fractions containing E2wt were pooled and concentrated with a 10 kD molecular weight cut off (MWCO) using Amicon Ultra Centrifugal Filter (Millipore). The retentate was loaded onto a Superdex200 gel filtration column (GE Healthcare) using Solubility Buffer 2.2 (1× PBS, 50 mM L-Glutamine (Sigma), 50 mM NaCl, 250 mM L-Arginine (Sigma)) and fractions containing the 1.5 MDa E2 60mer particles were concentrated to 1 mg/ml using the Ultra Centrifugal devices and then stored in Solubility Buffer 2.2 at −80°C.

Env(V3)-E2 and Env(min)-V3 proteins formed inclusion bodies in *E.coli* and were purified from the inclusion bodies, which were washed three times with Inclusion Body Wash Buffer (1 M guanidine hydrochloride (GuHCl), 50 mM NaCl, 1 mM DTT, 1× PBS, 10% glycerol, 0.5 M arginine, pH 7.4) before being dissolved in Unfolding Buffer (6 M GuHCl, 1 mM DTT, 1× PBS).

To produce the HIV-1 Env-E2 virus-like particles, E2wt was combined with the Env-E2 inclusion bodies at a 1∶1 molar ratio in Unfolding Buffer (6 M GuHCl, 1 mM DTT, 1× PBS) rocking at 4°C for a minimum of 3 h. The proteins were transferred to SnakeSkinT Dialysis Tubing, 10 K MWCO (Thermo Fisher Scientific) and subjected to step-down dialysis against 4 M, 2 M, 0 M GuHCl Refolding Buffers (4 M- 0 M GuHCl, 50 mM NaCl, 1× PBS, 10% glycerol, 0.5 M arginine, 0.5 mM reduced glutathione, 0.1 mM oxidized glutathione, pH 8.0). A final dialysis was performed in Solubility buffer 2.2 (1× PBS, 50 mM L-Glutamine, 50 mM NaCl, 250 mM L-Arginine, pH 7.4). Refolded soluble 60-mer virus-like particles were confirmed by gel filtration using the Superdex200 gel filtration column (GE Healthcare), and purity was assessed by SDS-PAGE and Western blot analysis. The purified VLPs were either stored directly at −80°C or subjected to LPS removal before storing at −80°C. LPS removal from protein samples utilized TritonX-114 (Sigma), as previously described [53], and the final particles were tested for endotoxin (LPS) using the Limulus Amebocyte Lysate (LAL) Assay (Lonza).

### SDS-PAGE and Western blot analysis

Expression, refolding, and identity of recombinant proteins were assessed by SDS-PAGE, western blot analysis, and ELISA. Samples were prepared as described above and resolved on Invitrogen NuPAGE 4–12% Bis-Tris mini-gels (Carlsbad, CA) under reducing conditions. For SDS-PAGE, gels were stained with SimplyBlue™ SafeStain (Invitrogen). For western blot analysis, proteins were transferred onto nitrocellulose paper (Invitrogen), blocked with Odessey blocking buffer (LI-COR Biosciences) overnight at 4°C. The following day, the blot was probed simultaneously with a 1∶8,000 dilution of rabbit sera specific for E2 and a 1∶20,000 dilution of the monoclonal human antibody 447-52D for 1 h at room temperature. After washing 5 times, secondary antibodies IRDye680 Goat anti-Rabbit and IRDye800 Goat anti-human (LI-COR Biosciences) were used at 1∶15,000. Membranes were scanned using the LI-COR Odyssey Infrared Imaging System (LI-COR Biosciences) to allow simultaneous two-color detection of E2 and the HIV-1 Envelope V3 region. Integrated intensities were used in conjunction with protein concentrations determined by Nanodrop to calculate protein purity and concentration. Purified particle preparations [wild type E2 and Env(V3)-E2] were tested for binding to antibody 447-52D after coating to ELISA plates at 200 ng/well using standard methods described previously [36]. No binding was seen to E2 wild type and equivalent binding to the V3 and min V3 particles was observed.

### Preparation of Helios Gene Gun DNA gold bullets

Plasmid DNA was precipitated onto 1 mm diameter gold beads, and bullets were prepared according to the manufacturer's instructions (Bio-Rad) and loaded with a total of 2 µg of DNA. To verify that the bullets were functional, COS-7 cells were transfected via Gene gun with the DNA carried by the gold beads. Cells were incubated at 37°C for 48 h and then fixed and stained for immunofluorescence using 0.5 µg/mL polyclonal primary antibody chimpanzee IgG derived from and HIV-infected animal (CHIVIG) and 1∶50 dilution of the secondary antibody FITC-conjugated goat anti-human IgG (Zymed). The presence of envelope-transfected cells was visualized by fluorescent microscopy.

### Antibody assays

Binding antibody responses in immunized mice and rabbits to E2-wt protein, V3 peptide and HIV-1 envelope antigen gp140 were measured by indirect ELISA as described previously [36]. Both endpoint and half-maximal methods were used to determine relative titers of antibodies, and both methods provided the same outcome of responses. Endpoint titers were most sensitive in our hands and necessary to observe response in mice; thus we report all endpoint titers to maintain consistency in evaluating antibody responses in mice and rabbits.

### Neutralization assays

Neutralization assays were performed using the single-cycle TZM-bl neutralization assay as described previously [54]. Neutralization activity of each sample was determined on the basis of the reduction in the *luc* reporter gene expression compared to that obtained in virus control wells containing virus and cells only {[(virus+cell)−(tested serum+virus+cell)]/virus+cell}×100 = % neutralization. Background control wells contained cells only. A pre-immune sera pool and the murine leukemia virus (MLV) were used as negative controls. A well-characterized immune serum was used as a positive control. Neutralization dose-response curves were fitted by non-linear regression and a final titer was informed as the reciprocal of the dilution of serum necessary to achieve 50% neutralization. Experiments were performed in parallel at ONPRC and at Duke University on key samples to compare results.

Detection of NAbs specifically directed to V3 peptide was performed as previously described [55]. Briefly, the TZM-bl neutralization assay was conducted with the inclusion of HIV-1 SF162 V3 peptide (PNNNTRKSITIGPGRAFYATGD) (Invitrogen, Carlsbad, CA) or the V3 scrambled peptide (PNNNTRKSIFYRGAPGITATGD) (Genscript, Piscataway, NJ) for 1 h, at a final concentration of 20 µg/mL with titrated rabbit sera (week 6, 14, 28 and 34), prior to 1 h incubation with 200 TCID_50_ of SF162 pV. Percent reduction in neutralization was calculated as: [1−(titer with V3 peptide)/(titer with V3 scrambled peptide)]×100.

### MHC class I dextramer staining and flow cytometric analysis

CD8^+^ T cells specific for IGPGRAFYA_311–318_ peptide were determined by MHC H-2Dd class I dextramer staining. Briefly, cells were transferred in 96-well round-bottom plates at density of 1.5×10^6^ cells/well and washed with PBS containing 5% FCS. Aliquots (10 µl) of PE-conjugated IGPGRAFYA_311–318_ H-2Dd dextramers or PE-conjugated H-2Dd control dextramers (Immudex, Dako, Copenhagen, Denmark) were then added to the cells. The plates were gently vortexed and incubated in the dark at room temperature for 10 min. Control dextramer that does not recognize mouse CD8^+^ T cells was used to assess non-specific staining. Cells were then stained with FITC-conjugated anti mouse-CD8 mAb for 20 min in the dark at 2–8°C. After two washes with buffer, stained cells were resuspended in PBS and a minimum of 50,000 live, CD8-positive, gated events were acquired and analyzed by flow cytometry using a FACSCanto (BD Biosciences). Results are expressed as the percentage of CD8^+^ cells that are positive for the MHC I/peptide dextramer.

### Intracellular cytokine IFNg staining (IFNg-ICS)

To identify antigen-specific IFN**g**-secreting CD8^+^ T cells, we performed intracellular cytokine IFNγ staining with Cytofix/Cytoperm Leucoperm™ kit (AbD Serotec, Kidlington, UK), as previously described by Caivano et al [36]. Briefly, 1.5×10^6^ splenocytes per well were stimulated with 10 µg/ml of synthetic IGPGRAFYA_311–318_ peptide in U-bottom 96-well plates. The cultures were incubated at 37°C in a 5% CO_2_ incubator for 5 h with 10 µg/ml of the secretion inhibitor Brefeldin-A (Sigma-Aldrich). The IFNγ release induced by 30 ng/ml of Phorbol 12-myristate 13-acetate (PMA, Sigma-Aldrich) plus 1 µg/ml of ionomycin (Sigma) was used as a positive control. After the stimulation period, cells were washed with PBS containing 1% FCS, subsequently incubated for 15 min at 4°C with FITC-conjugated anti-mouse CD8 mAb for surface staining, followed by fixation with Cytofix/Cytoperm™ solution for 15 min at 4°C. The surface-stained cells were then permeabilized with 1X Perm/Wash™ solution and stained intracellularly by incubation with PE-conjugated anti-mouse IFNγ mAb for 30 min at room temperature. The cells were finally washed twice with PBS and acquired on FACSCanto (BD Biosciences).

### Antibodies and synthetic peptides

The following monoclonal antibodies (mAb) were used for FACS analysis: fluorescein isothiocyanate (FITC)-conjugated anti-mouse CD8 (clone 53-6.7, Biolegend, San Diego, Ca); phycoerythrin (PE)-conjugated anti-mouse IFNγ (clone XMG1.2, eBioscience, Hatfield, UK). MAb 447-52D to HIV-1 V3 was obtained through the NIH AIDS Research and Reference Program, Division of AIDS, NIAID, NIH from Susan Zolla-Pazner. Polyclonal antibody CHIVIG was described previously [56]. For FACS analysis the synthetic IGPGRAFYA_311–318_ peptide, a 9-mer peptide corresponding to residues 311–318 of the V3 loop of HIV-1 Envelope glycoprotein, was purchased from PRIMM srl (Naples, Italy). For peptide competition assays, the HIV-1 SF162 V3 peptide (PNNNTRKSITIGPGRAFYATGD) and the V3 scrambled peptide (PNNNTRKSIFYRGAPGITATGD) were purchased from Invitrogen (Carlsbad, CA) and Genscript (Piscataway, NJ), respectively.

### Statistics

Statistical analyses were performed using either the unpaired Student t-test, the Mann-Whitney test (comparison between two groups) and Analysis of Variance (ANOVA, for comparison among three or more groups). To compare NAb levels along the different immunization regimens, the area under the NAb titer curve (AUC) minus baseline was calculated and divided by the duration of each regimen, and significance was determined using the Mann-Whitney test. In all cases differences were considered statistically significant when P<0.05 (and represented as * when P<0.05, and ** when P<0.01).
